# An overview of agents and treatments for PDGFRA-mutated gastrointestinal stromal tumors

**DOI:** 10.3389/fonc.2022.927587

**Published:** 2022-08-31

**Authors:** Yingchao Sun, Lei Yue, Pengfu Xu, Weiling Hu

**Affiliations:** ^1^ Department of Gastroenterology, Sir Run Run Shaw Hospital, Medical School, Zhejiang University, Hangzhou, China; ^2^ Department of Gastrointestinal Surgery, Taizhou Hospital, Zhejiang University, Taizhou, China; ^3^ Institute of Gastroenterology, Zhejiang University (IGZJU), Hangzhou, China; ^4^ Zhejiang University Cancer Center, Hangzhou, China

**Keywords:** PDGFRA mutation, targeted therapy, avapritinib, ripretinib, crenolanib, gastrointestinal stromal tumors (GIST)

## Abstract

Platelet-derived growth factor receptor A (PDGFRA) mutations occur in approximately 10–15% of gastrointestinal stromal tumors (GISTs). These tumors with PDGFRA mutations have a different pathogenesis, clinical characteristics, and treatment response compared to tumors with receptor tyrosine kinase protein (KIT) mutations (60–70%). Many clinical studies have investigated the use of tyrosine kinase inhibitors mainly in patients with KIT mutations; however, there is a lack of attention to the PDGFRA-mutated molecular subtype. The main effective inhibitors of PDGFRA are ripretinib, avapritinib, and crenolanib, and their mechanisms and efficacy in GIST (as confirmed in clinical trials) are described in this review. Some multi-targeted tyrosine kinase inhibitors with inhibitory effects on this molecular subtype are also introduced and summarized in this paper. This review focuses on PDGFRA-mutated GISTs, introduces their clinical characteristics, downstream molecular signaling pathways, and existing resistance mechanisms. We focus on the most recent literature that describes the development of PDGFRA inhibitors and their use in clinical trials, as well as the potential benefits from different combination therapy strategies.

## Introduction

Gastrointestinal stromal tumors (GIST) are sarcomas that mostly derive from precursors of the interstitial cells of Cajal (ICC). Although GISTs are the most common sarcoma of the GI tract, they are rare, with an incidence of only 10–15 patients per million per year (1, 2). GISTs are a heterogeneous group of tumors, including multiple molecular subtypes, with various activating oncogene mutations, such as receptor tyrosine kinase protein (KIT; approximately 60%–70%) and platelet-derived growth factor receptor A (PDGFRA; approximately 10%–15%) (3, 4), each of which present with different pathological mechanisms, clinical characteristics, and treatment response (5). PDGFRA is the second most mutated oncogene in GIST, and the annual incidence of PDGFRA-mutated GISTs is < 3 cases per 1 million individuals (6). PDGFRA-mutated GISTs can derive from telocytes and they show an epithelioid pattern (7, 8). PDGFRA-mutated GISTs are mostly located in the stomach (15–18%), followed by the small intestine (5–7%) (9). Around 15% of GISTs are wild-type GIST, which have no mutations in either KIT or PDGFRA, but have other genetic alterations, such as in the succinate dehydrogenase (SDH) gene family, RAS gene family, proto-oncogene B-Raf (BRAF), neurofibromatosis type 1 (NF1), phosphatidylinositol-4,5-bisphosphate 3-kinase catalytic subunit alpha (PIK3CA), gene fusions involving ETS variant transcription factor 6 (ETV6)-neurotrophic tyrosine receptor kinase 3 (NTRK3) or fibroblast growth factor receptor 1 (FGFR1), or other rare driver gene mutations (10–13).

**Figure 1:**
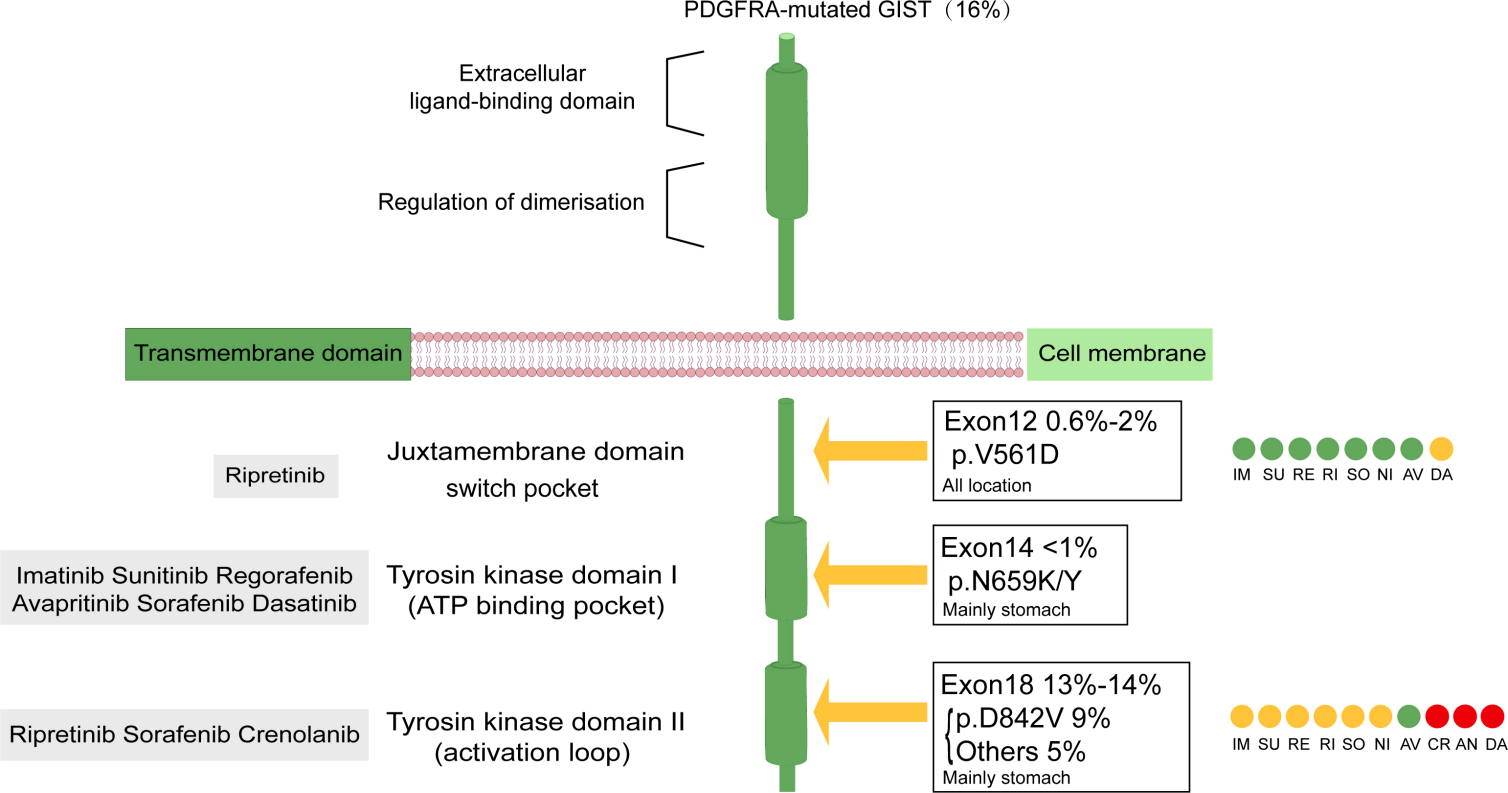
Structure of PDGFRA transmembrane tyrosine kinase receptor. Graphical representation of PDGFRA transmembrane tyrosine kinase receptors with frequency and localization of mutations found in advanced GIST. Gray boxes represent the site of action of the drug. Drug sensitivity of primary and secondary mutations in PDGFRA-mutated GISTs was distinguished by color: green indicates sensitive, yellow indicates mainly sensitive, red indicates in vitro test proved to be valid, but the clinical trial still had no definite result.CR Clinical trials in PDGFRA D842V population still ongoing; DA AN in vitro trial Valid but no clinical trial; NI specifically involving PDGFRA in vitro demonstrated activity against exon 12, diminished activity against D842V. AV, avapritinib; IM, imatinib; RE, regorafenib; RI, ripretinib; SU, sunitinib: SO, sorafenib; CR, crenolanib, DA, dasatinib; Ni, nilotinib; AN, anlotinib.

PDGF was first discovered during platelet activation. Its receptor has two similar structures, PDGFRA and PDGFRB, which undergo intracellular activation during transport of the exocytic pathway and are subsequently secreted (14, 15). PDGFRA, similar to KIT, encodes the receptor tyrosine kinase (RTK), is located on chromosome 4q11-q12 (16), and is associated with many physiological processes of human growth and development. PDGFR consists of an extracellular ligand-binding region, a single transmembrane-spanning region, and an intracellular tyrosine kinase domain (17). This dual-switch mechanism carefully regulates cellular kinase activity by control of kinase conformation. Mechanistically, switch control of kinase conformation is mediated by phosphorylation of one or more switch amino acids that turn the kinase “on” or “off” (18). Most primary and secondary resistance mutations in PDGFRA are located within conformation-controlling switch regions embedded in the intracellular kinase domain. Primary resistance is more frequent in the activation loop (6). Secondary resistance is commonly located in the ATP-binding domain (exon 14) or activation loop (exon 18) (7, 19) (**Figure 1**). Mutations in PDGFRA are mainly found in exons 18 and 12 and rarely occur in exon 14 (4). Exon 18 encodes the activation loop and represents approximately 80% of the PDGFRA-mutated GISTs. A single D842V mutation, substitution of aspartic acid to valine, creates a missense mutation that confers resistance to imatinib, sunitinib and regorafenib. D842V is the most common exon 18 mutation, and it is detected in 62.6% of PDGFRA-mutated tumors (3, 4, 20). Exon 12, encoding the juxta membrane domain, is mutated in approximately 0.6% – 2% of GISTs, and < 1% of PDGFRA mutations will occur in exon 14 (encoding the ATP-binding domain). Studies have shown that novel tyrosine kinase inhibitors (TKIs), avapritinib and ripretinib, target the PDGFRA D842V mutation in GISTs and provide objective responses and long-term tumor control (21). Two clinical trials investigating the use of crenolanib in GISTs (NCT01243346 and NCT02847429), which is also used for targeting the PDGFRA D842V mutation, are still ongoing.

**Figure 2:**
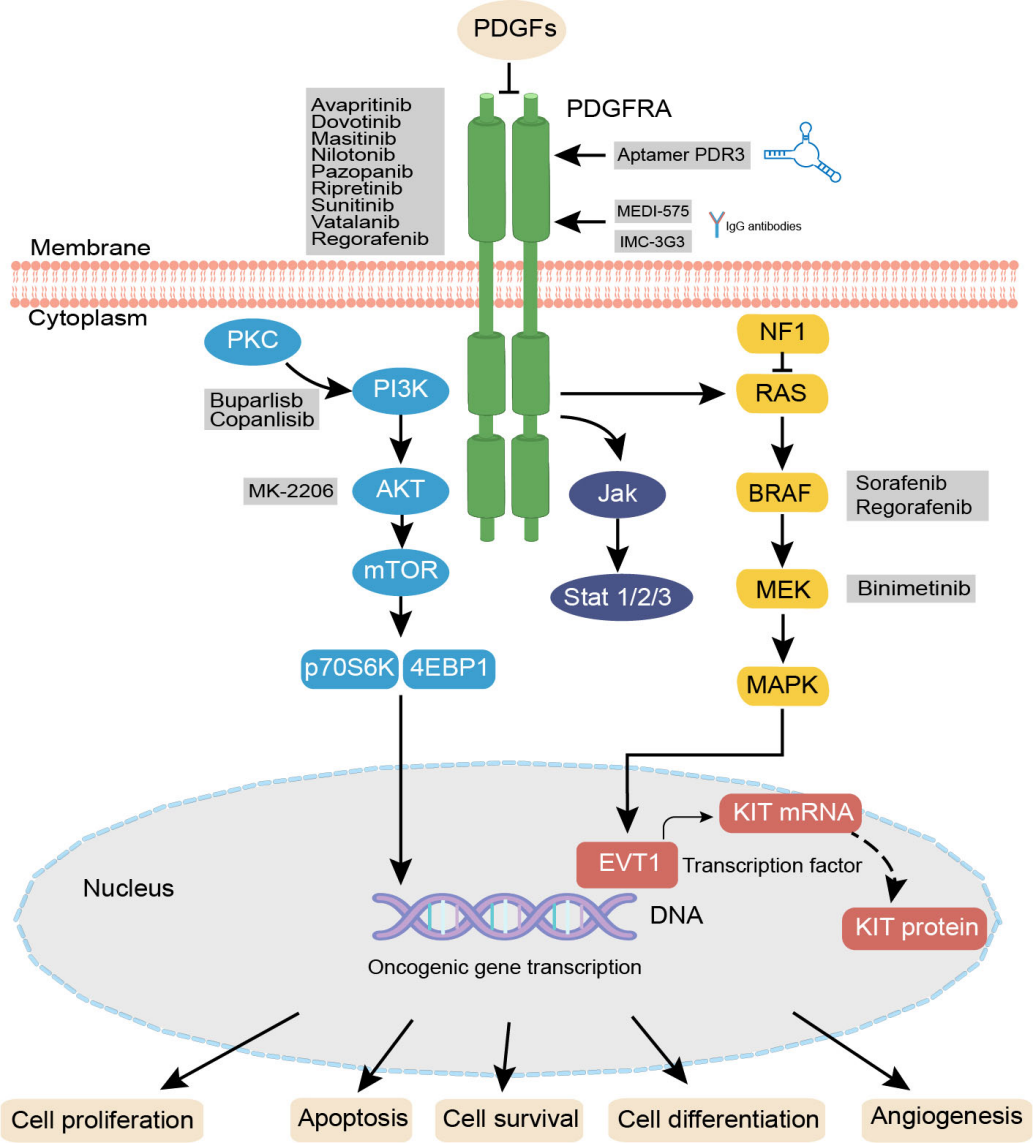
PDGF/PDGFRA signaling pathways and agent treatments. In PDGFRA mutaled GIST. PDGF isoforms bind to the related PDGFRs, initiate distinct receptor dimerization and phosphorylation, thereby activating various signaling pathways including the RAS-MEK-MAPK, PI3K-mTOR, JAK/STAT, and Notch pathway PDGFR antibodies such as INC-3G3, MEDI-575 and the specific RNA aptamer PDR3 can selectively bind to PDGFRA, after binding, these antibodies or aptamers can inhibit the downstream pathways. Activation or inhibition of these pathways influences cellular proliferation, angiogenesis, and apoptosis. PDGF, platelet-derived growth factor; PDGFR, platelet-derived growth factor receptor; PKC, protein kinase C; PI3K, phosphati-dylinositol 3 kinase; AKT/PKB, protein kinase B; JAK, Janus kinase; STAT, signal transducers and activators of transcription; ERK, extracellular signal-regulated kinase; MAPK, mitogen-activated protein kinase; PDR3, the specific RNA aptamer.

The PDGFR signaling pathway is an important RTK pathway that is associated with physiological activity in a variety of tumors (22). PDGFRs are transmembrane glycoprotein dimer molecules that initiate dimerization and phosphorylation after binding to the PDGF ligand, thereby activating various downstream signaling pathways, such as phosphatidylinositol 3 kinase (PI3K)/protein kinase B (AKT/PKB) pathway, mitogen-activated protein kinase (MAPK)/extracellular signal-regulated kinase (ERK) pathway, Janus kinase (JAK)/signal transducers and activators of transcription (STAT) pathway, and the Notch pathway (23, 24). Inhibition of PDGFR suppresses cancer proliferation, metastasis, invasion, and angiogenesis, and improves the antitumor effects of cancer drugs (25, 26). Some novel therapeutic strategies have emerged based on the PDGFR pathway for cancer treatment (**Figure 2**).

This review will update the current knowledge on characteristics of PDGFRA-mutated GISTs and further discuss the clinical management of this subtype. After a brief introduction of first-, second-, and third-line TKIs for GIST treatment, we provide an overview of drugs based on other mechanisms, with a focus on new generation TKIs and future combination strategies.

## First generation TKIs for treatment of PDGFRA-mutated GISTs: Imatinib, sunitinib, and regorafenib

During the past two decades, GIST has become a successful paradigm for the rational development of molecular targeted agents, based on the poor efficacy of cytotoxic chemotherapy (27) and the satisfactory improvement of prognosis by targeted therapy (28, 29).

Imatinib, an oral small molecule TKI which is a competitive inhibitor of the ATP binding site of KIT and PDGFRA, is the first-line drug for patients receiving initial treatment for advanced GISTs (30). A series of clinical trials have demonstrated the efficacy of imatinib (28, 29, 31). For advanced disease, regardless of mutation type, a daily dose of 400 mg provided a median progression free survival (mPFS) of 18 months. Approximately 45% of patients had a partial response, up to 5% had a complete response, and 32% had stable disease (27). Imatinib is active against non-D842V PDGFRA-mutated GISTs, but lacks efficacy against tumors with the D842V mutation, with significant differences in objective response rates between the two subtypes (32). A total of 58 patients with PDGFRA mutations were included in an international survey study, of whom 55% carried PDGFRA-D842V mutations (33). None of the patients with the D842V mutation achieved a response, and the mPFS was 2.8 months. For patients with other PDGFRA mutations, the mPFS was 28.5 months and overall response rate was 36% (33).

Sunitinib is approved as a second-line treatment for advanced GIST following progression on imatinib, or for patients with intolerance to imatinib. Like imatinib, sunitinib targets KIT and PDGFR, however, sunitinib may be effective in imatinib-resistant GISTs through its broader binding profile and affinity. MPFS was 6.8 months in patients receiving sunitinib and 1.6 months in those on placebo (34). *In vitro* studies have shown that the efficacy of sunitinib on PDGFRA exon 18 activation loop mutations is poor (35). A non-interventional retrospective analysis (NCT00094029) explored the correlation between PDGFRA mutation status and clinical benefit in patients treated with sunitinib (36). In this study, only 12 patients (5%) had a PDGFRA mutation, thus, the available data is too limited to draw conclusions on the efficacy of sunitinib on survival outcomes in patients. In a Korean cohort, patients with PDGFRA exon 18 mutations treated with sunitinib exhibited stable disease as the best response. Patients with D842V mutations had a 25% probability of stable disease after 24 weeks (37).

Regorafenib has the broadest kinase inhibitory activity among approved agents, which is a competitive inhibitor of the ATP-binding site for PDGFR, vascular endothelial growth factor receptor 1–3 (VEGFR1–3), TEK, KIT, RET, RAF1, BRAF, and FGFR (38). Regorafenib is approved as a third-line treatment for advanced GIST after progression on imatinib and sunitinib (39). In a case report of a patient with PDGFRA D842V mutated GIST, treatment with regorafenib resulted in prolonged response, and 20 months after treatment onset, the patient is still under treatment and maintaining a partial response (40). However, there is limiting evidence relating mutational status to regorafenib response. The mPFS and overall response rate (ORR) of patients with PDGFRA mutations treated with early generation TKIs are summarized in **Table 1**.

**Table 1:** Response rates and PFS of approved agents for PDGFRA-mutated GISTs.

Agents	Mutation status, if known	Response rate (%)	MPFS(m)	Refs
Imatinib	D842V	0	2.8-3.8	(33)
	non-D842V	36-71	28.5-29.5	(33) (32)
Sunitinib	–	0	2.8	(36)
Regorafenib	D842V	1/1	>20	(40)
Ripretinib	- -	9 -^a^	6.3 6.8	(41) (42)
Avapritinib	D842V KIT or nonD842V PDGFRA	91 17	34 4.2	(21) (43, 44)

a: In the INTRIGUE study, after excluding the population with kit exon 11 or 9 mutations, in the other KIT and PDGFRA mutant populations, the mPFS was 6.8 months, but the ORR value was not statistically analyzed. mPFS, median progression‐free survival; ORR, overall response rate; TEAEs, treatment related adverse events.

There are currently no phase II trials that have specifically included patients with PDGFRA mutations and there is no evidence on the efficacy of sunitinib or regorafenib in PDGFRA mutations available from phase III trials. Imatinib, sunitinib and regorafenib are type II multi-kinase inhibitors, which bind to the ATP-pocket of PDGFRA only in the inactive formation, and they have limited activity against activation loop mutations (45). Recently approved next-generation TKIs, avapritinib and ripretinib, were specifically developed to address this issue.

## Next-generation TKIs in GIST: Ripretinib and avapritinib

### Ripretinib

Ripretinib was authorized by the FDA in March 2020 as a fourth-line or later-line treatment for GIST patients previously treated with three or more kinase inhibitors, including imatinib. Ripretinib is a novel type II switch-controlled kinase inhibitor that regulates both the kinase switch pocket and the activation loop (45), and the dual mechanism of action maintains KIT and PDGFRA in an inactive conformation independent of the primary and secondary mutation type, thereby inhibiting downstream signaling (46).

The phase III INVICTUS trial (NCT03353753) evaluated ripretinib using a dose of 150 mg daily in a fourth-line or later-line setting compared with placebo in patients who were refractory or intolerant to at least all three approved TKIs (41). In 129 patients, mPFS was 6.3 months in ripretinib-treated patients compared to one month in the placebo group, with a hazard ratio of 0.15 (95% CI 0.09–0.25). Ripretinib and placebo groups showed different response rate and median overall survival (OS) (9% vs 0% and 15.1 months vs 6.6 months, respectively). This trial met the primary end point, and disease stabilization was 47% at 12 weeks.

A second phase III trial, INTRIGUE (NCT03673501), is evaluating the safety and efficacy of ripretinib versus sunitinib as a second-line therapy (47). A total of 453 patients with GIST were enrolled in this study, where 226 patients received continuous dosing of 150 mg of ripretinib and 227 patients received continuous dosing of 50 mg of sunitinib (4-week on and 2-week off). Although the study did not meet its primary endpoint, progression-free survival (PFS) in ripretinib treated patients was not superior those treated with sunitinib, but there were significant advantages in ORR and safety profiles (42). In all population, mPFS was similar in patients treated with ripretinib compared to those treated with sunitinib: 8.0 months vs 8.3 months (HR = 1.05, 95% CI 0.82 to 1.33). The ORR for patients with a KIT exon 11 mutation was higher in those treated with ripretinib compared to those treated with sunitinib (23.9% vs 14.6%, respectively), however, sunitinib treatment improved PFS for patients with KIT exon 9 mutations.

The overall safety profile of ripretinib was favorable, with most side effects being low grade and manageable. In the INVICTUS trial, the most common grade 1/2 non-hematologic adverse events (AEs) occurred in more than 20% of patients and included alopecia (49%), myalgia (27%), nausea (25%), fatigue (24%), hand-foot skin reaction (HFSR) (21%), and diarrhea (20%). The most common grade 3/4 AEs included lipase increase (5%), hypertension (4%), fatigue (2%) and hypophosphatemia (2%) (48). In the INTRIGUE STUDY, the safety profile of ripretinib was improved compared to that of sunitinib, with a lower incidence of grade 3/4 AEs (26.5% vs 55.2%). For grade 3/4 AEs with an incidence of ≥2%, the incidence of these events was also lower in the ripretinib group than in the sunitinib group (42).

Dose escalation can be an alternative treatment option after disease progression, which has been effective in patients receiving imatinib (49). Additionally, there was a study that explored the efficacy of 150 mg of ripretinib twice daily in patients with advanced GIST used as a second, third or later line of therapy (50). In this study, 142 patients were included, and 67 patients received 150 mg of ripretinib twice daily after disease progression, which provided benefit across all lines of therapy. The mPFS was 5.6, 3.3, and 4.6 months in second-, third- and fourth-line therapy, respectively. The rate of partial metabolic response was 35.5%. Similarly, in the Phase III INVICTUS study, 43 patients received a twice daily dose of 150 mg of ripretinib 150 mg after disease progression. The mPFS was 3.7 months and the safety profile was acceptable (51).

### Avapritinib

In contrast to early generation TKIs, avapritinib was specifically designed as a potent and highly selective type I inhibitor of mutations affecting the activation loop (encoded by exon 17 in KIT and exon 18 in PDGFRA) (45, 52). Avapritinib was approved by the FDA in 2020 based on the phase I/II trial results (21) for advanced or metastatic PDGFRA-mutated GIST, including the exon 18 D842V mutation.

The safety and efficacy of avapritinib was evaluated in 2 clinical trials, NAVIGATOR (NCT02508532) and VOYAGER (NCT03465722). In the NAVIGATOR study, a first-in-human phase I clinical trial, defined the starting dose as 300 mg and the maximum tolerated dose as 400 mg daily (21). The efficacy results are impressive, as the overall response rate was 91% at a dose of 300 mg daily (51/56 patients), the clinical benefit rate was 98% (55/56 patients), and the mPFS was 34.0 months (43). In this study, avapritinib had an acceptable safety profile, and AEs were generally grade 1 or grade 2. Most AEs that occurred were similar to those observed from other TKIs, such as edema, nausea, vomiting, and diarrhea. Other AEs of special interest were identified, including cognitive effects (48%, including memory impairment (29%), confused mental status (7%), encephalopathy (1%), and other cognitive disorders (11%)) and intracranial hemorrhage (2%) (53). However, the long-term cognitive side effects for avapritinib remain unknown.

The activity of avapritinib was compared to that of regorafenib in GIST as a fourth-line treatment in a phase III trial (VOYAGER), which randomized metastatic GIST patients to either avapritinib (n=240) or regorafenib group (n=236). Early top-line data indicated that avapritinib did not demonstrate an improvement over regorafenib in terms of PFS, the primary end point of the study. The mPFS was reported to be 4.2 months for avapritinib and 5.6 months for regorafenib, disease control rates were 41.7% vs 46.2%, and the overall response rate was 17.1% vs 7.2%, respectively (54).

Although avapritinib remains as the best treatment option for patients with advanced GIST with the PDGFRA D842V mutation, studies have revealed that secondary resistance may still develop in patients. Resistance mutations are observed within PDGFRA exons 13, 14, and 15, and these secondary PDGFRA mutations cause V658A, N659K, Y676C, and G680R substitutions that impair avapritinib binding (55).

## Other TKIs beyond standard therapy for GIST

Drug resistance is a typical clinical phenomenon in cancer therapy, therefore, there are new clinical studies being conducted to address this dilemma. **Table 2** lists clinical trials of novel next-generation TKIs (ripretinib and avapritinib) and other non-FDA approved agents that target PDGFRA mutations in metastatic or locally advanced GISTs.

**Table 2:** Overview of clinical trials for agents targeting PDGFRA mutations in metastatic or locally advanced GIST.

Agent	Target of agent	Trial	phase	Purpose	Clinical Trial	Estimated finished time
Ripretinib	a novel type II switch-controlled kinase inhibitor	INVICTUS	III	Assess ripretinib as a fourth-line or later treatment	NCT03353753	finished
		INTRIGUE	III	Compare ripretinib to Sunitinib in patients refractory to Imatinib	NCT03673501	finished
Avapritinib	Inhibition of KIT and PDGFRA activation loop	NAVIGATOR	I	Evaluate avapritinib as first-line treatment	NCT02508532	finished
		VOYAGER	III	Compare avapritinib to regorafenib in patients previously treated with Imatinib and other TKI(s)	NCT03465722	finished
Dasatinib	Inhibition of KIT, PDGFR, BCR-ABL and SRC		II	Assess Dasatinib as first-line treatment	NCT00568750	finished
			II	Assess Dasatinib as third-line treatment	NCT02776878	finished
Ponatinib	Inhibition of KIT, PDGFR, FGFR, VEGFR, FLT3	POETIG	II	Assess Ponatinib as second-line treatment (pretreatment with Imatinib)	NCT03171389	2020.09(but still recruiting
Crenolanib	Inhibition of PDGFRA (including D842V)	–	III	Crenolanib for patients with PDGFRA D842V mutation	NCT02847429	2021.08 still no results)
Lenvatinib	Inhibition of KIT, PDGFRA, FGFR1-4, VEGFR1-3, and RET	LENVAGIST	II	pretreatment with Imatinib and Sunitinib	NCT04193553	2022.03
DS6157a	anti-GPR20 antibody-drug conjugate	–	I	pretreatment with Imatinib	NCT04276415	2022.05
Pimitespib	Inhibition of heat shock protein 90	–	III	pretreatment with all three TKIs approved	JapicCTI-184094	finished
		–	II	pretreatment with all three TKIs approved	JapicCTI-163182	finished
Dovitinib	Inhibition of KIT, PDGFR, VEGFR 1-3, FGFRs 1-3, FLT3	DOVIGIST	II	Assess Dovitinib as second-line treatment	NCT01478373	finished
Vatalinib	Inhibition of KIT, PDGFR, and VEGFR	–	II	pretreatment with Imatinib or both Imatinib and Sunitinib.	NCT00117299	finished
PLX9486+Sunitinib	target 2 complementary conformational states of kinase	–	Ib/IIa	assess whether combination is associated with broad mutation coverage and global disease control.	NCT02401815	finished
Imatinib+ buparlisib	a PI3K pathway inhibitor+ type II TKI	–	Ib	pretreatment with Imatinib and Sunitinib	NCT01468688	finished
Regorafenib+avelumab	TKI combined with immunotherapy	REGOMUNE	II	Evaluate effect of combination for solid tumors	NCT03475953	2022.12

### Dasatinib

Dasatinib is a small molecule tyrosine kinase inhibitor with multiple targets including KIT, BCR-ABL, PDGFR, and nonreceptor kinases (SRC family) (56). Structurally, it differs from imatinib and sunitinib as it can bind to the ATP binding pocket regardless of receptor conformation (active and inactive states) (57, 58). A preclinical study has shown that GIST cells harboring the PDGFRA D842V mutation respond to dasatinib, where they exhibited reduced cell proliferation after exposure to dasatinib *in vitro* (59).

The efficacy and safety of dasatinib as a first-line therapy for GIST was explored in a single-arm phase II trial (60). The FDG-PET/CT response rate at 4 weeks was 74% and mPFS was 13.6 months. Grade 4 AEs occurred in 5% of patients and grade 3 AEs occurred in 48% of patients, most commonly involving gastrointestinal or pulmonary events. These results demonstrate that imatinib offers more benefit and favorable toxicity profile compared to dasatinib. Another single-arm phase II trial investigated the activity of dasatinib as a third-line therapy in 58 GIST patients who failed to respond to imatinib and sunitinib. Preliminary results showed that the 3-month PFS rate was 53.4% and the median OS was 14.0 months (61).

### Crenolanib

Crenolanib is an orally available, highly specific inhibitor of PDGFR family members, and has a 25-fold higher affinity for PDGFR than KIT (62). Crenolanib was significantly more potent than imatinib in inhibiting the kinase activity of PDGFRA *via* the activation loop of exon 18: D842I, D842V, D842Y, DI842-843IM, and deletion I843. *In vitro* experiments showed that crenolanib was 100 to 150-fold more potent than imatinib against D842V, with an IC50 of approximately 10 nmol/L (63). In KIT-mutant GIST, crenolanib-mediated inhibition of PDGFRA disrupted the KIT-ERK-ETV1-KIT signaling loop by inhibiting ERK activation (64).

Crenolanib has been tested in both phase I and Ib clinical studies and it was found to be well tolerated in patients (65). Currently, there is a phase II trial using 140 mg of crenolanib twice daily in patients with advanced GIST with D842-related mutations and deletions (including D842V mutations) in the PDGFRA gene. Similarly, a randomized phase III trial (NCT02847429) used 100 mg of crenolanib three times daily or matching placebo for patients with advanced or metastatic GIST exhibiting the PDGFRA D842V mutation. The clinical trials are still ongoing and the results have not been disclosed.

### Nilotinib

Nilotinib is a selective TKI targeting KIT, PDGFR and BCR-ABL (66). Both *in vitro* proliferation and *in vivo* studies indicated that nilotinib exhibited anti-tumor activity against the PDGFRA V561D mutation but significantly reduced efficacy against PDGFRA D842V mutation (67). Nilotinib has been used in 18 clinical trials, including 15 completed trials, two not recruiting, and one active trial (68). Nilotinib was compared with imatinib in a randomized phase III trial as a first-line therapy (69). The trial did not meet the primary endpoint of improvement in PFS. Molecular analysis showed that for patients with other mutations (excluding KIT 11, 9, and wild type), OS rates were comparable in both arms (69). A phase III trial explored the efficacy of nilotinib in patients with advanced GIST after previous imatinib and sunitinib failure (70). The patients were randomized in a 2:1 ratio to either 400 mg of nilotinib twice daily or a best supportive care group (best support only, or with imatinib/sunitinib). Local investigator-based intent-to-treat (ITT) analysis showed a significant increase in mPFS in the nilotinib group (119 days vs 70 days). Median OS was higher in patients with nilotinib (332 days vs 280 days). However, the PFS based on blinded central radiology review (primary endpoint) was not significantly different, therefore, further development of nilotinib as a third-line treatment for GIST was discontinued.

### Anlotinib

Anlotinib, a multi-targeted TKI, is characterized as a highly selective and potent c-KIT and PDGFR inhibitor (71). Anlotinib has a broad spectrum of antitumor activity against GIST with D842V, D816H, V560G and V654A mutations both *in vitro* and *in vivo* (72). The toxicity profile of anlotinib in the treatment of a variety of advanced tumors was consistent with that reported for sorafenib, sunitinib, and regorafenib (73, 74). In a phase I clinical trial using anlotinib for advanced refractory solid tumors, the most common serious AEs were hand-foot skin reactions, hypertension, fatigue, and lipase elevation (71).

A single-arm, multicenter phase II trial (NCT04106024) is currently exploring the efficacy and safety of anlotinib in patients with advanced GIST refractory to imatinib. The clinical trial plans to enroll 60 participants who will be treated with 12 mg of anlotinib once daily for two weeks, followed by a one week break, and ending with three weeks as a course of treatment. The primary outcome measures is PFS up to 18 months. The trial is currently fully recruited and the results remain undisclosed.

### Dovitinib

Dovitinib is a multikinase inhibitor of KIT, PDGFR, VEGFR 1-3, FGFRs 1-3, and FLT3 (75). In a phase I study, dovitinib administration to a patient with GIST resulted in disease control for eight months after failed response to imatinib and sorafenib (76). A phase II trial (DOVIGIST, NCT01478373) evaluated the antitumor activity of dovitinib as a second-line therapy in patients with GIST who were refractory to or intolerant of imatinib, including a total of 38 patients, of which 21 patients had KIT mutations and three patients had PDGFRA mutations. The ORR was 2.6% (1 of 38 patients), all three patients with PDGFRA mutation had stable disease, and the PFS was 4.6 months (77). In a phase II clinical trial of dovitinib as a third-line therapy, dovitinib showed modest anti-tumor activity and manageable toxicity. The study included 30 patients with failed response to imatinib or sunitinib, with a disease control rate of 13% at 24 weeks, a partial response in one patient (3%), stable disease in the other 21 patients, a mPFS of 3.6 months, and a median OS of 9.7 months (78).

### Sorafenib

Sorafenib is similar to sunitinib, as it is a multikinase inhibitor with selectivity for KIT, PDGFRA, BRAF and FLT-3 (79, 80), however, it is not approved for treatment of GIST*. In vitro* studies demonstrated that sorafenib could inhibit imatinib-resistant PDGFRA mutations that occur in exon 14 (encoding the ATP-binding pocket) and exon 18 (encoding the activation loop), except for the substitution of PDGFRA codon 842 (79). A retrospective study has also investigated sorafenib in patients being resistant or intolerant to imatinib or sunitinib and showed a mPFS of 6.4 months after sorafenib treatment (81). Two phase II trials investigated sorafenib as a third-line treatment for patients with advanced GIST. One trial including 31 patients demonstrated a mPFS of 4.9 months and a disease control rate of 36% (82). Another trial performed with 38 patients reported that the disease control rate was 68%, and mPFS was 5.2 months after treatment (83). A case report showed that a patient with deletion of codon p.I843_D846del (located at PDGFRA exon 18), who was highly sensitive to sorafenib, responded in a dose-related manner. This patient had been treated with sorafenib for 12.5 years and still had no signs of recurrence (84). No phase III trials utilizing sorafenib for treatment of GIST have been conducted.

### Lenvatinib

Lenvatinib is a type I TKI that also acts as an inhibitor of several RTKs, targets KIT, PDGFRA, FGFR, VEGFR, and RET, and has been approved for various advanced cancers (85, 86). The IC50 of lenvatinib ranged from 29 to 39 nM in *in vitro* assays (87, 88). It may have profound effects on tumor cell migration and invasion by inhibiting FGFR and PDGFR (89). A prospective, randomized, double-blind, multicenter trial (NCT04193553) aims to evaluate the efficacy and safety of lenvatinib in patients with GISTs who had previously failed imatinib and sunitinib (90). In this trial, 37 patients are treated with a continuous daily oral dose of 24 mg of lenvatinib and they are provided with best supportive care. The expected PFS is 1.5 months in the control group and 3.0 months in the lenvatinib group (HR=0.5), and the study is expected to be completed in March 2023.

### Heat shock protein 90 (HSP90) inhibitor pimitespib (TAS116)

HSP90 is a molecular chaperone that assists many proteins, including KIT and PDGFRA, and maintains the structure and activity of certain key signaling proteins by folding and stabilizing proteins (91, 92). *In vitro* studies have shown that HSP 90 inhibitors can inhibit PDGFRA and attenuate downstream protein phosphorylation (59).

In a single-arm phase II trial (93), the oral HPS90 inhibitor TAS-116 was used in patients who were refractory to imatinib, sunitinib and regorafenib. MPFS was 4.4 months, the progression-free rate at 12 weeks was 74%, and 85% of patients had stable disease after 6 weeks. The most common AEs were gastrointestinal disorders and ocular AEs, and all other grade 3 or higher AEs resolved after dose modification.

In a phase III trial (JapicCTI-184094), pimitespib was compared with placebo in the treatment of patients with advanced GIST refractory to all three approved TKIs (94). The primary endpoint of the study was met and the results showed that the mPFS was 2.8 months vs 1.4 months, respectively, resulting in a 49% reduction in the risk of disease progression or death. This encouraging result suggests that HSP90 inhibitors are a potential novel therapy for patients with advanced GIST.

### Azd3229

AZD3229 is a highly potent and selective small molecule inhibitor of KIT/PDGFRA that inhibits a wide range of primary and secondary mutations in GISTs without VEGFR2 inhibition (95, 96). *In vitro* assays and xenograft models showed that AZD3229 is more potent and selective than other approved agents, including avapritinib, and AZD3229 is 15–60 times more potent than imatinib in inhibiting primary mutations in KIT (96). Therefore, AZD3229 has the potential to be a best-in-class inhibitor for clinically relevant KIT/PDGFRA mutations in GIST.

### Anti-GPR20 antibody-drug (DS-6157a)

Previous transcriptional studies have suggested that GIST cells may be rich in a novel gene target called G-protein coupled receptor 20 (GPR20) (97), which is expressed in more than 80% of GIST specimens, although GPR20 expression levels are lower in PDGFRA mutant GISTs (98). DS-6157a is an anti-GPR20 antibody-drug conjugate with a novel tetrapeptide-based linker and a derivative of the DNA topoisomerase I inhibitor exatecan derivative. In GIST xenograft models, including GIST models resistant to imatinib, sunitinib, and regorafenib, anti-GPR20 resulted in anti-tumor activity, and showed a favorable kinetic and safety profile. However, further clinical trials are still needed to verify the safety and efficacy of this novel agent.

## Agent combination based on different strategies

Polyclonal resistance is a significant obstacle in GIST treatment, as a single drug is insufficient to target all resistant KIT mutations. Clinical trials have studied TKI therapies with different target inhibitors for tumors with polyclonal secondary mutations to address tumor heterogeneity. Several strategies based on therapeutic combinations aim to overcome the resistance mechanisms. A list of different combination based strategies is provided in **Table 3**.

**Table 3:** Agent combination based on different strategies in the treatment of Imatinib-resistant GIST.

Purpose	Strategy	Agent Combination	Clinical Trial.gov identifier
Enhancing complementary Inhibition against KIT mutations	TKI + TKI	Ib/IIa: PLX9486+Sunitinib	NCT02401815
		Sunitinib +Regorafenib	NCT02164240
Preventing KIT downstream pathway	TKI + inhibition of PI3K	Ib: Imatinib + Buparlisib	NCT01468688
		I: Imatinib+BYL719	NCT01735968
	TKI+ inhibition of mTOR	I: Imatinib +Everolimus	NCT01275222
	TKI + inhibition of AKT	Imatinib + MK2206	*In vitro* and *in vivo* study
		II: Imatinib+Perifosine	NCT00455559
Preventing adaptation to KIT inhibition	TKI + inhibition of FGFR	Ib: BGJ398 + Imatinib	NCT02257541
Maintaining stability and activity of KIT	TKI + inhibition of HSP90	I: Imatinib + Onalespib	NCT01294202
Inhibiting immune escape	TKI + immunotherapy	II: Regorafenib+ Avelumab	NCT03475953
		Ib: Dasatinib + Ipilimumab	NCT01738139
		II: Axitinib + Avelumab	NCT04258956
		I/II: Imatinib + PDR001	NCT03609424
Future area	TKI + inhibition of BRAF/TKI + inhibition of MET TKI + inhibition of apoptosis inducer		

Sunitinib and regorafenib have complementary inhibitory characteristics against KIT mutations, and clinical trials with polyclonal secondary mutant tumors have utilized TKI therapy as a rapid alternating therapy. In the SURE project (NCT02164240), an open-label phase I/II trial, 3 days of sunitinib were followed by 4 days of regorafenib in imatinib-refractory advanced GISTs (99). Four of the 13 patients included had stable illness, and the overall mPFS was 1.9 months (99). Another promising drug combination with complementary efficacy is PLX9486 (a selective type I KIT inhibitor targeting activating loop mutations) and sunitinib. A phase 1b/2a clinical trial (NCT02401815) in 39 patients was conducted to determine whether it is associated with wide mutation coverage, and it demonstrated activity with a mPFS of 12.1 months and an 80% clinical benefit rate (100).

The second strategy is to act on signaling pathways designed to prevent KIT downstream pathway to enhance apoptosis, such as the combination of imatinib with other critical targets (RAS/MAPK or PI3K/mTOR). In GIST patients who had previously failed treatment with imatinib and sunitinib, a Phase 1b study (NCT01468688) using the PI3K inhibitor buparlisib in combination with Imatinib was conducted to assess the clinical profile of the combination (101). However, compared to currently accessible treatments, this combination offered no significant benefit. No partial or full responses were observed, therefore further development of this combination was not pursued. Another Dose-finding Study (NCT01735968) of a Combination of Imatinib and BYL719, PI3K inhibitor in the Treatment of 3rd Line GIST Patients is conducted, but there are no results disclosed (102).

Tumor adaptation to KIT/PDGFRA inhibition leads to apoptosis evasion, and this antiapoptotic response is sustained over time by FGFR- and c-MET–mediated MAPK pathway reactivation (103, 104). It is plausible that GIST follows the same principles of chronic myeloid leukemia, where growth factor receptors are inhibited by a MEK-dependent negative feedback that is released upon BCR-ABL TKI inhibition (105). Binimetinib, a reversible inhibitor of mitogen-activated extracellular signal-regulated kinase 1 (MEK1) and MEK2 activity, is approved for the treatment of unresectable or metastatic melanoma with BRAF V600E or V600K mutations. Binimetinib is currently being investigated in a phase 1 study in patients with advanced GISTs in combination with pexidartinib (NCT03158103) (106). Another phase II trial (NCT0199379) was designed to test the efficacy and safety of binimetinib plus imatinib as a first-line treatment for GIST, which met the primary endpoint and showed good efficacy and manageable toxicity (107). Dual targeting of the GIST lineage-specific master regulators, ETV1 and KIT, by MEK and KIT inhibitors, respectively, may enhance clinical efficacy of these agents, and more studies should be conducted to explore this combination.

The fourth strategy is TKI combined with immunotherapy. REGOMUNE (NCT03475953) is a single arm, multicentric phase II trial investigating the safety and efficacy of regorafenib (160 mg daily for 3 weeks with 1 week rest) in combination with avelumab (once every 2 weeks) in patients with various solid tumors. At present, the results of the GIST subgroup have not been disclosed, but synergistic anti-tumor effect has been shown in biliary tract tumors and colorectal cancer (108, 109).

## Future of advanced GIST treatment

Although the combination of imatinib and other targeted inhibitors failed to meet primary clinical outcomes, these studies were conducted in imatinib-resistant GIST, in which case imatinib is unlikely to bind to KIT secondary mutants, and therefore, would have limited efficacy. Future research should consider combinations of other approved TKIs (sunitinib, regorafenib, ripretinib and avapritinib) with other targeted inhibitors. Furthermore, crenolanib is a highly specific inhibitor of PDGFR, including the D842V mutation subtype, thus, further studies should be conducted to explore its combinatorial effects with other agents. Innovative forms of combination therapy such as intermittent or drug rotation regimens can also be explored to achieve effective doses while minimizing overlapping toxicities (99).

Notch signaling is a conserved developmental pathway known to play a critical role in the development of multiple tumors (110, 111). In soft tissue sarcoma, including GIST, multiple studies reported deregulated Notch expression (112). A previous study reported the tumor suppressor effects of the Notch pathway in GISTs *via* negative feedback with the oncogene KIT and may lead the development of new therapeutic strategies for GISTs patients (113). Only one clinical trial utilizing LY3039478, an oral selective Notch inhibitor, was conducted in patients with GIST to assess its safety, pharmacokinetics, and antitumor efficacy (114). Given the role of Notch inhibitors in the treatment of other cancers, more clinical trials should be done in the future to investigate the effect of notch inhibitors, including gamma-secretase complex inhibitors, as well as anti-Notch2/3 antibody treatments, for the treatment of GIST.

Activation of the PI3K/AKT/mTOR pathway, a key downstream pathway of KIT/PDGFRA signaling, has been shown to be a crucial survival pathway in imatinib-resistant GISTs (115). Many mutations in genes of this signaling pathway, including IGF1R, MTOR, TSC1, FLT4, TSC2, IRS1, INSR, and BRCA1, may mediate resistance to imatinib or other KIT inhibitors (116). Several clinical trials targeting PI3K/AKT/mTOR signaling are currently being investigated as promising targeted therapy strategies for GIST (117). In a phase I/II clinical trial, the oral mTOR inhibitor everolimus demonstrated efficacy in GIST refractory to imatinib and sunitinib, with 37% of patients remaining progression-free for at least 4 months and 36% of patients achieved stable disease (118). Another mTOR inhibitor, sirolimus, has shown promise in a small number of GIST patients with PDGFRA-D842V mutations when combined with TKIs such as imatinib (119). Concurrent inhibition of the two critical KIT-downstream pathways is regarded as a very attractive approach. To investigate the ideal combination dose in the future, additional clinical trials are required.

Studies have shown that adaptive and innate immune cells are present in the GIST tumor microenvironment, which suggests immunotherapy may be a potential future treatment for GIST. Many studies indicate that D842V-mutated tumors exhibited a significant enrichment of immune-related genes and immune cells, such as CD3+, CD8+, and CD68+ cells (120). Compared to KIT mutant GISTs, PDGFRA mutant GISTs express significantly higher levels of chemokines, such as CXCL14, and these tumors could exhibit HLA binding (121). Immunotherapeutic agents used in clinical trials include anti-PD-1 (nivolumab, spartalizumab, pembrolizumab, avelumab, and PDR001)/PD-L1 molecules and ipilimumab (targeting CTLA-4). Nowadays, only one clinical trial each of pembrolizumab and ipilimumab has been completed (122, 123), showing limited activity.

Tumor heterogeneity is another major concern in cancer therapy. Liquid biopsies, combined with sequencing techniques, have the potential to predict TKI treatment sensitivity by capturing circulating tumor DNA (ctDNA) and cells (124). Future efforts in laboratory research should be applied to decipher lineage-specific KIT dependence (125). Parallel efforts should point to studies of high-throughput synthetic lethal screens, for example, the discovery that CDC37 is a critical HSP90 cofactor for oncogenic expression of KIT provides a promising strategy (126). Preclinical and clinical evidence supports the exploration of novel treatment modalities aimed at blocking various mechanisms of resistance or adaptation. Integrating clinical-genomic data and generating robust preclinical models will be the backbone of successful future GIST research.

## Conclusion

In conclusion, two decades of active translational and clinical research have demonstrated the paradigm of GISTs as targeted therapies. Multi-kinase inhibitors, such as sunitinib, regorafenib, dasatinib, nilotinib, anlotinib, dovitinib, pazopanib, sorafenib, and lenvatinib, are currently preferred above over other treatment strategies. Efforts were subsequently made to develop highly selective kinase inhibitors, including avapritinib and crenolanib, to improve kinome selectivity. Proteasome inhibitors such as bortezomib and histone deacetylase inhibitors such as SAHA or panobinostat have also been postulated as potential treatments for GISTs. Activation of the PI3K/AKT/mTOR pathway has been shown to be a critical survival pathway, and multiple clinical trials of mTOR inhibitors and PI3K inhibitors in the treatment of GISTs are also being carried out. The majority of current efforts are focused on reducing the hazards brought on by polyclonal heterogeneity in imatinib-refractory GIST. The necessity to research novel agents or, more likely, treatment combinations intended to jointly block multiple resistance mechanisms is being supported by preclinical and clinical evidence more and more.

## Author contributions

YS and WH devised the main conceptual ideas and outline. All authors contributed to the article and approved the submitted version.

## Conflict of interest

The authors declare that the research was conducted in the absence of any commercial or financial relationships that could be construed as a potential conflict of interest.

## Publisher’s note

All claims expressed in this article are solely those of the authors and do not necessarily represent those of their affiliated organizations, or those of the publisher, the editors and the reviewers. Any product that may be evaluated in this article, or claim that may be made by its manufacturer, is not guaranteed or endorsed by the publisher.

## Abbreviations

Platelet-derived growth factor receptor A (PDGFRA), gastrointestinal stromal tumors (GISTs), tyrosine kinase protein (KIT), interstitial cells of Cajal (ICC), succinate dehydrogenase (SDH) gene family, RAS gene family, proto-oncogene B-Raf (BRAF), neurofibromatosis type 1 (NF1), phosphatidylinositol-4,5-bisphosphate 3-kinase catalytic subunit alpha (PIK3CA), gene fusions involving ETS variant transcription factor 6 (ETV6)-neurotrophic tyrosine receptor kinase 3 (NTRK3) or fibroblast growth factor receptor 1 (FGFR1), receptor tyrosine kinase (RTK), phosphatidylinositol 3 kinase (PI3K)/protein kinase B (AKT/PKB) pathway, mitogen-activated protein kinase (MAPK)/extracellular signal-regulated kinase (ERK) pathway, Janus kinase (JAK)/signal transducers and activators of transcription (STAT), vascular endothelial growth factor receptor 1–3 (VEGFR1–3), median progression free survival (mPFS), overall response rate (ORR), adverse events (AEs), G-protein coupled receptor 20 (GPR20), mitogen-activated extracellular signal-regulated kinase 1 (MEK1).
